# Efficiency of QuEChERS approach for determining 52 pesticide residues in honey and honey bees

**DOI:** 10.1016/j.mex.2016.05.005

**Published:** 2016-05-18

**Authors:** Pau Calatayud-Vernich, Fernando Calatayud, Enrique Simó, Yolanda Picó

**Affiliations:** a Environmental and Food Safety Research Group (SAMA-UV), Research Center on Desertification (CIDE, UV-CSIC-GV), Faculty of Pharmacy, University of Valencia, Av. Vicent Andrés Estellés s/n, 46100 Burjassot, Valencia, Spain; b Agrupación de Defensa Sanitaria Apícola apiADS, Ctra. Montroi-Turís, 46193 Montroi, Valencia, Spain

**Keywords:** QuEChERS (quick, easy, cheap, effective, rugged and Safe), Solvent extraction, SPE (solvent phase extraction), QuEChERS, solid phase extraction (SPE), solvent extraction, honey, honey bee, pesticide, LC–MS/MS

## Abstract

A comparison between QuEChERS and other pesticide extraction procedures for honey and honey bee matrices is discussed. Honey bee matrix was extracted by solvent based procedure whereas solid phase extraction was the protocol for the honey matrix. The citrate buffered QuEChERS method was used for both matrices. The methods were evaluated regarding cost (equipment and reagents), time, accuracy, precision, sensitivity and versatility. The results proved that the QuEChERS protocol was the most efficient method for the extraction of the selected pesticides in both matrices.

• QuEChERS is the most economical and less time-consuming procedure.
• SPE and solvent-based extraction procedures show equivalent recoveries to QuEChERS.
• QuEChERS can be used to extract pesticide residues from both matrices.

## Graphical abstract

**Figure:**
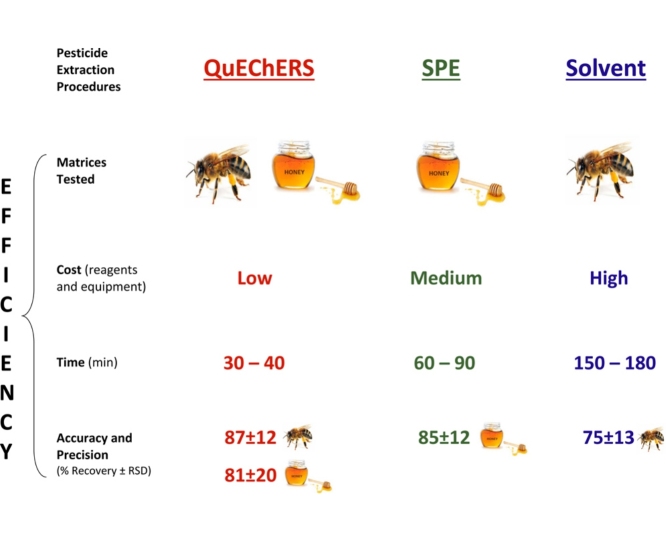

## Method details

### QuEChERS approach for the extraction of pesticide residues in honey and honey bee matrices [1], [2], [3].

1) Weigh 5 g of honey or honey bees into 50 mL centrifuge tubes and add 7.5 mL of water, 10 mL of acetonitrile, 6 g of MgSO4 and 1 g of NaCl. Homogenize the mixture immediately and then, centrifuge for 5 min at 300 rpm.
2) Put 2 mL of the supernatant into another 15 mL centrifuge tube containing 50 mg C18, 50 mg PSA, and 150 mg MgSO4. Vortex the mix and centrifuge it for 5 min at 3000 rpm.
3) Finally, filter the supernatant using a PTFE 13 mm × 0.22 μm into the autosampler vials for LC–MS analysis.

### Solvent approach for the extraction of pesticide residues in honey bee matrix [4].

1) Weigh 5 g of honey bees and pound thoroughly in a glass mortar. When homogenized place in a 250 mL flask and mix it vigorously for 10 min with 20 mL of acetone.
2) Filter the mixture in a Kitassato flask through a Buchner funnel of 13 cm with a paper filter packed with a layer of Celite 545 (5–10 mm) and wash the filter cake with 20 mL of acetone.
3) Prepare 100 mL, with 1% weight/volume (w/v) ammonium chloride and 2% volume/volume (v/v) ortophosphoric acid (85%) and add it to the filtrate. Allow it to stand for 30 min with occasional stirring and then filter with Celite 545.
4) After filtration, dilute the sample with 200 mL of 2% aqueous sodium chloride (w/v) and extract twice with 100 mL of dichloromethane.
5) Pass the resultant organic phase through a filter containing anhydrous sodium sulfate and evaporate it to dryness in a rotary evaporator at 35 °C.
6) Dissolve the extract obtained from the honey bee samples in acetone, up to 2 mL, for GC analysis. For LC–MS determination, evaporate to dryness a 1-mL aliquot of the previous extract using a gentle stream of nitrogen and then dissolve it in the same volume of methanol.

### Solid phase extraction (SPE) approach for the extraction of pesticide residues in honey matrix [5].

1) Weigh honey (1.5 g) and mix it with 30 mL of hot water (<80 °C). Agitate by a stir bar for 10 min.
2) Pre-condition an Oasis HLB cartridge [poly (divinylbenzene-co-*N*-pyrrolidone)] with 5 mL of methanol and 5 mL of Milli-Q water.
3) Pass the mix through the cartridge at a flow rate of 10 mL min^−1^.
4) Rinse the cartridge with 5 mL of Milli-Q water.
5) Dry the cartridge under vacuum for 15 min.
6) Elute the retained pesticides by passing 10 mL of methanol–dichloromethane (3:7).
7) Evaporate the eluate to 0.5 mL using a gentle steam of nitrogen.
8) Then, transfer it into 1-mL volumetric flask with methanol, obtaining a final extract in 100% methanol.

## Liquid chromatography–mass spectrometry

Inject 5 μL of the extract in the LC–MS/MS according to the conditions already reported [1] and detailed below.

Ionization and fragmentation settings were optimized by direct injection of pesticide standard solutions. MS/MS was performed in the SRM mode using ESI in positive mode. For each compound, two characteristic product ions of the protonated molecule [M+H]^+^ were monitored, the first and most abundant one was used for quantification, while the second one was used as a qualifier. Collision energy and cone voltage were optimized for each pesticide (Table 1). Nitrogen was used as collision, nebulising and desolvation gas. The ESI conditions were: capillary voltage 4000 V, nebulizer 15 psi, source temperature 300 °C and gas flow 10 L min^−1^. In order to maximize sensitivity, dynamic MRM was used, with MS_1_ and MS_2_ at unit resolution and cell acceleration voltage of 7 eV for all the compounds.

## Quality assurance/quality control (QA/QC)

In order to compare QuEChERS to other routine procedures, methods were validated according to the European Union Guideliness [6]. Furthermore, the main elements of uncertainty as the amount of sample used for a determination, the recovery value of the analytical procedure and the repeatability of determinations for a true sample [7], were considered through the validation process (for detailed information of the validation parameters, see Supplementary material Table S1 and S2).

**Fig. 1:**
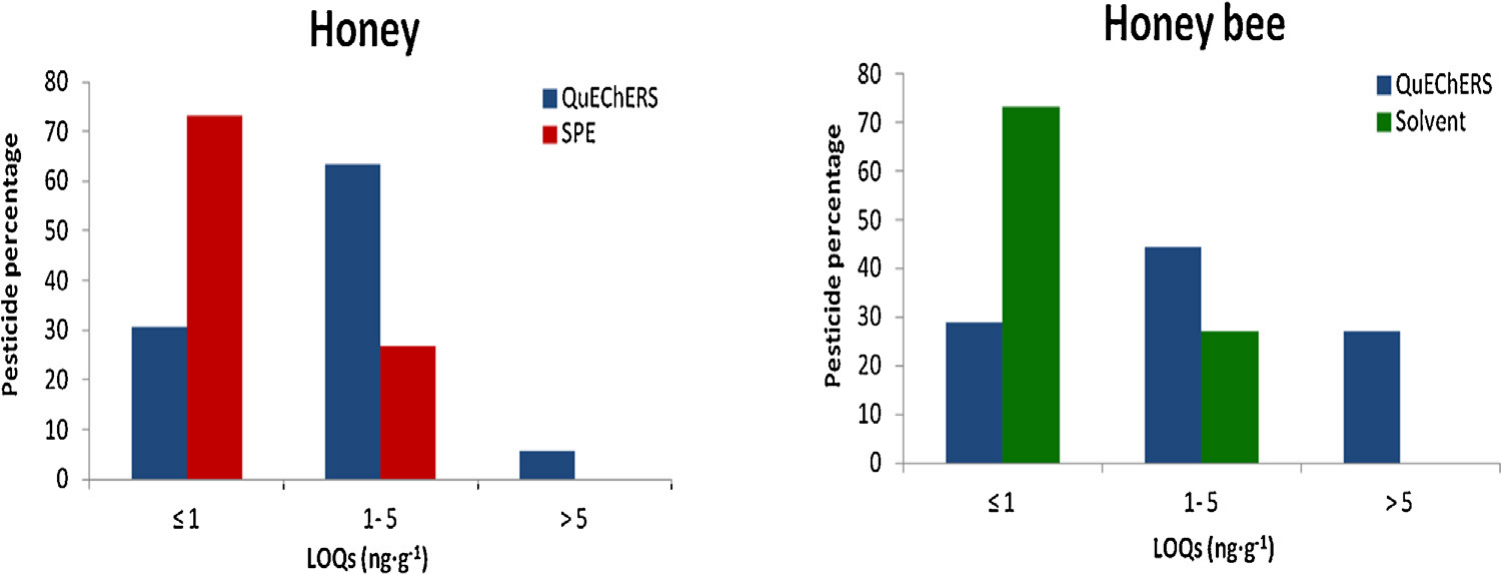
Limits of quantitation (LOQs) of QuEChERS, SPE and solvent methods in honey and honey bee matrices.

The sensitivity of the method was estimated by establishing the limits of quantification (LOQs) (Fig. 1). The LOQs were determined in pure solvent and in spiked honey and honey bees samples. LOQs were calculated as the lowest concentration or mass of the analyte that has been validated with acceptable accuracy by applying the complete analytical method. LOQs were from 0.2 to 10 ng g^−1^ and from 0.03 to 10 ng g^−1^ for honey and honey bee matrices respectively. Solvent and SPE methods were slightly more sensitive than QuEChERS approach.

**Fig. 2:**
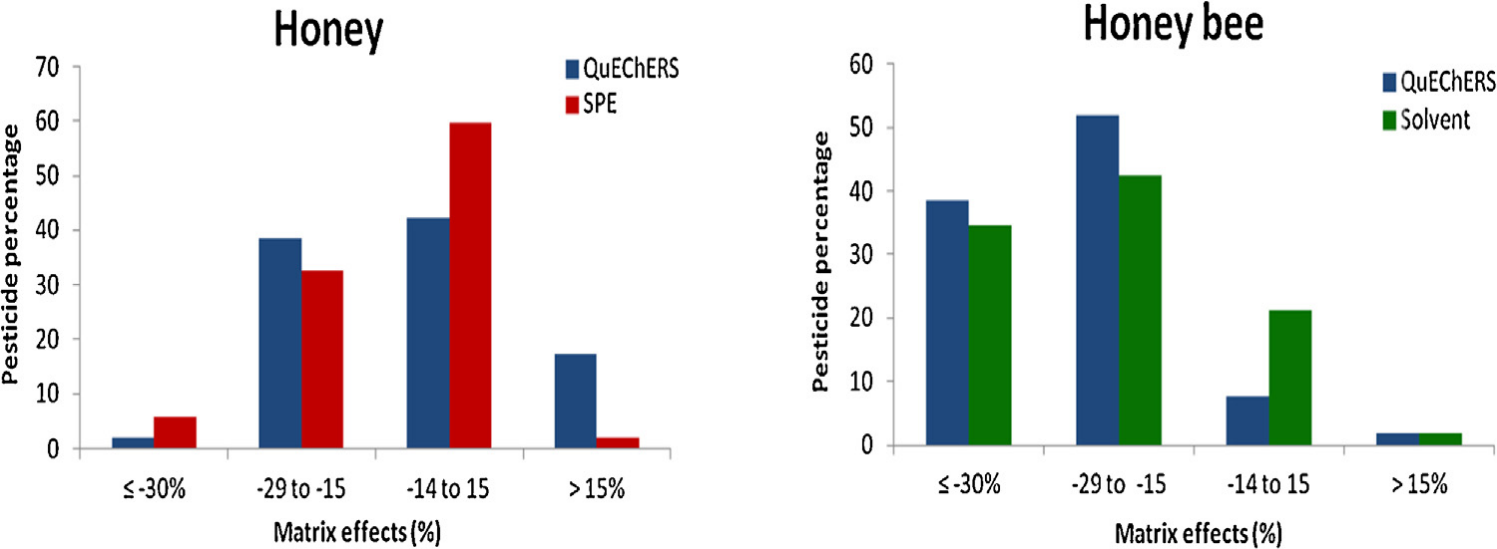
Matrix effects of QuEChERS, SPE and solvent methods in honey and honey bee matrices.

Matrix effects were evaluated by comparing the slope of the previous calibration curve and the slope of that prepared in the extract of honey or honey bee matrix with six concentration levels of standard solutions (Fig. 2). Matrix effects were mostly suppressive in both matrices and ranged from −60 to 50 and from −60 to 35% in honey and honey bee matrices, respectively.

**Fig. 3:**
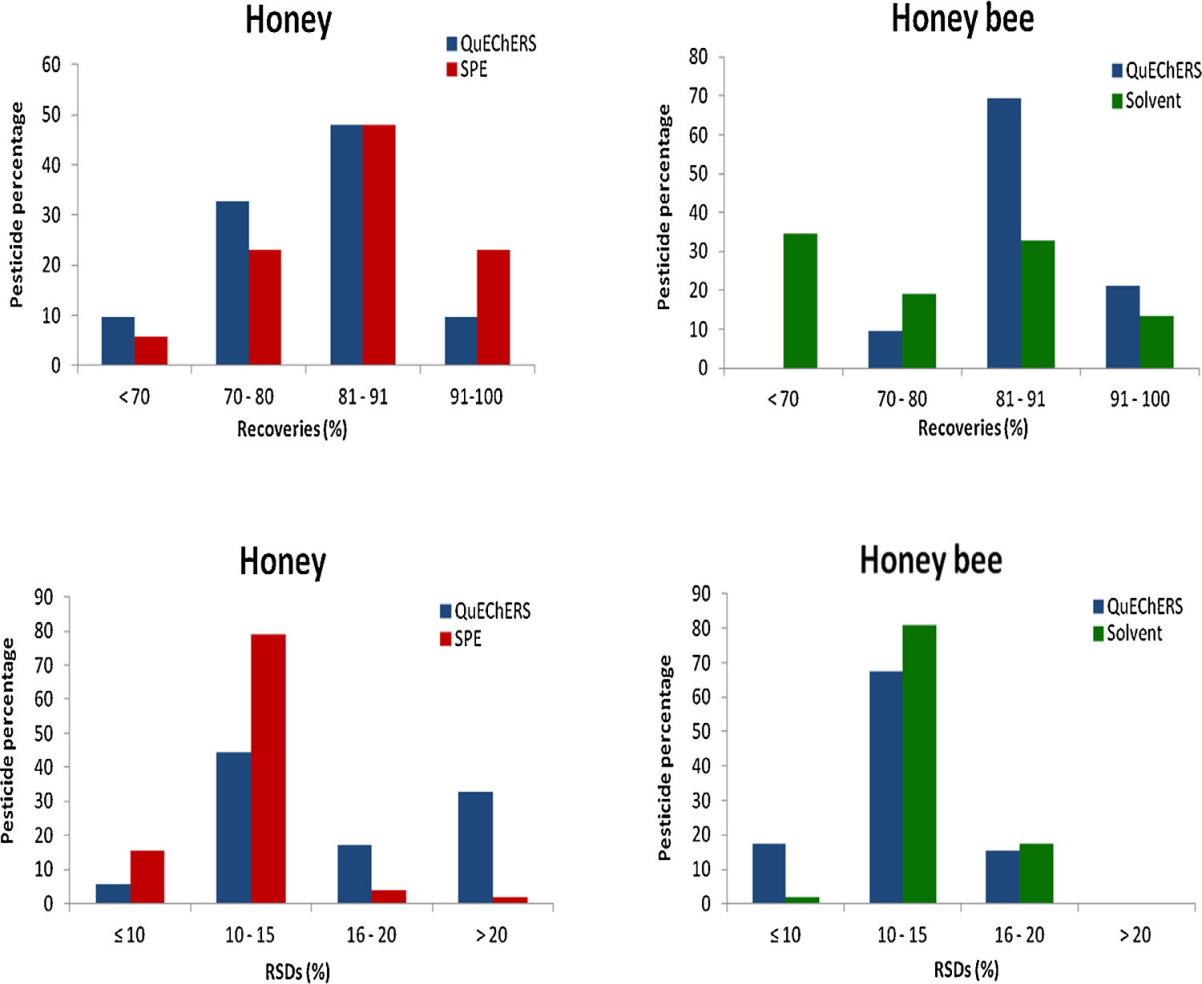
Accuracy (Recoveries) and precision (RSDs) validation parameters of QuEChERS, SPE and solvent methods in honey and honey bee matrices.

Mean recovery (as accuracy) and relative standard deviation (as precision) were evaluated by spiking the samples at the LOQ and 10 x LOQ, with a minimum of 5 replicates (Fig. 3). Recovery values of honey bee matrix were from 34 to 96%, whereas RSDs were in all cases <20%. Honey matrix showed recoveries that ranged from 30 to 96% and RDS were <20% except for 17 compounds that were from 21 to 42%. QuEChERS approach showed better results than solvent method in the honey bee matrix while SPE was slightly better both in accuracy and precision than QuEChERS extraction procedure for honey.

## Additional information

The use of pesticides in agricultural cropping systems is often discussed as a factor influencing honey bee health [1]. Furthermore, honey, which is considered a healthy natural product, can be contaminated during its production from both agricultural and beekeeping practices [8], [5]. The development of extraction procedures able to process samples in an economic way is crucial.

This paper presents some of the currently applied sample preparation methods for the separation and pre-concentration of pesticides in honey and honey bee samples. The composition of honey and honey bees is very different but both are complex matrices. In order to achieve an accurate and reliable analytical result, an efficient pre-concentration/separation step is usually required prior to determination, even when such a sensitive detection method as LC–MS/MS is used.

From an analytical point of view, honey can be considered as a highly concentrated sugar solution (mostly fructose). Then, after water dilution it can be extracted using protocols similar to those applied to water as SPE. The protocol described here requires a medium cost in reagent and equipment because the SPE sorbents involve a high cost. The extraction of a sample requires between 60 and 90 min, being evaporation the step that takes more time. The performance of the method provides the best sensitivity and lower matrix effects.

On the contrary, honey bees are rich in lipids and proteins, requiring most sophisticated and extensive sample preparation methods. Traditional methods as the solvent approach are long, tedious and require high amounts of expensive organic solvents [4]. Considering the use of reagents and equipment this method has high cost, requires between 150 and 180 min to process a sample and provides recoveries slightly lower for more polar pesticides

**Fig. 4:**
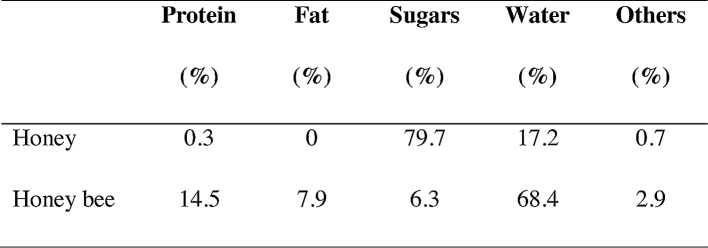
Honey and honey bee composition (%) [12], [13], [14].

The results pointed out that QuEChERS approach is used in many different matrices as hive products (beeswax, pollen, honey, honey bee) [9], [3], [10]. Honey and honey bee composition (Fig. 4) evidence the versatility of the QuEChERS method compared to other extraction procedures as those used in the present work. Appropriate results in terms of specificity, selectivity, accuracy and sensitivity, low cost and quickness make QuEChERS a suitable procedure for determining pesticides in less studied hive matrices as royal jelly and propolis. Furthermore, QuEChERS approach meets important components of green analytical chemistry [11] due to its small amounts of solvent needed compared to the traditional methods.

## Figures and Tables

**Table 1:** Dynamic MRM conditions used for LC–MS/MS determination of pesticide residues.

Target Pesticide	*t_R_*^a^ (min)	Δ *t_R_*^b^	Precursor Ion	SRM_1_^c^	Frag^d^ (V)	CE^e^ (V)	SMR_2_^f^	Frag^d^ (V)	CE^e^ (V)	SMR_2_/SRM_1_ (%) (%RSD)^g^
Acetamiprid	2.67	3.21	223	126	111	22	56	111	14	37.4 (12)
Acetochlor	10.07	2	270	224	120	10	148	120	10	46.8 (22)
Alachlor	10.07	2	270	238	80	15	162	80	10	50.4 (13)
Atrazine	6.52	2.63	216	132	120	15	174	120	20	17.3 (14)
Atrazine-desethyl	2.54	2.5	188	146	120	15	104	121	24	29.1 (15)
Atrazine-desisopropyl	1.75	2.08	174	96	120	15	132	120	15	78.6 (13)
Azinphos-ethyl	10.16	1.71	346	97	80	20	137	80	32	83.5 (12)
Azinphos-methyl	8.17	1.24	318	125	80	8	132	80	12	85.4 (11)
Buprofezin	14.5	1.1	306	201	120	10	116	120	15	64.6 (13)
Carbendazim	4.54	4.74	192	160	95	17	132	95	25	11.4 (14)
Carbofuran	4.37	2.91	222	123	120	10	165	70	15	98.0 (9.3)
Carbofuran-3-hydroxy	1.85	2.48	255	163	70	5	220	70	15	90.8 (9)
Chlorfenvinphos	11.74	1.61	359	155	120	10	127	120	15	63.8 (11)
Chlorpyriphos	15.33	2.23	350	350	92	13	198	97	13	78.6 (14)
Coumpahos	14.05	2.15	363	335	134	10	307	134	10	24.8 (10)
Diazinon	11.77	1.89	305	169	128	17	153	128	21	66.3 (12)
Dichlofenthion	14.68	2	315	259	120	10	287	120	5	44 (11)
Dimethoate	2.06	2.59	230	199	80	10	171	80	5	45.3 (12)
Diuron	7.5	1.25	233	72	120	20	160	120	20	3.2 (13)
DMF	5.14	4.5	150	132	111	10	107	111	15	41.6 (16)
Ethion	14.88	1.23	385	199	80	5	171	80	15	35.3 (11)
Fenitrothion	10.03	1.18	278	125	140	15	109	121	12	95.5 (12)
Fenthion	11.51	1.83	279	247	114	5	169	114	13	76.6 (10)
Fipronil	13.33	2.85	437	368	150	15	290	150	25	21.8 (11)
Flumethrin	18.53	1.85	527	267	50	10	239	50	10	48.3 (18)
Fluvalinate	18.11	1.81	503	208	50	10	181	50	26	73.4 (10)
Hexythiazox	15.11	1.15	353	228	120	20	168	120	10	67.4 (9)
Imazalil	11.4	1.71	297	159	120	20	201	120	15	56 (14)
Imidacloprid	1.61	1.96	256	209	80	10	175	80	10	75 (11)
Isoproturon	6.83	2.37	207	72	120	20	165	120	10	16.8 (12)
Malathion	9.36	1.96	331	99	80	10	127	80	5	98.5 (4)
Methiocarb	8.64	1.93	226	121	80	5	169	80	10	66.6 (11)
Metholachlor	10.49	2.04	284	252	120	15	176	120	10	10 (14)
Molinate	9.41	1.98	188	126	80	20	55	80	10	61.7 (11)
Omethoate	1.06	2.67	214	125	80	5	183	80	20	72.3 (12)
Parathion-ethyl	11.11	1.91	292	236	88	4	264	88	8	45.5 (13)
Parathion-methyl	8.17	1.5	264	125	120	20	232	110	5	34.5 (13)
Prochloraz	12.08	1.91	376	308	80	10	266	80	10	14.3 (9)
Propanil	8.6	2.01	218	162	120	20	127	120	15	92.4 (11)
Propazine	8.74	2	230	146	120	15	188	120	20	93.3 (14)
Pyriproxyfen	14.78	1.33	322	227	120	10	185	120	10	36.1 (12)
Simazine	4.53	1.76	202	124	120	20	132	120	20	93.8 (12)
Tebuconazole	13.82	2.87	308	125	95	25	70	95	21	6.6 (11)
Terbumeton	10.98	2.89	226	170	95	17	114	95	25	13.8 (14)
Terbumeton-desethyl	6.69	3.76	198	142	90	13	86	90	25	31.7 (12)
Terbuthylazine	11.1	3.01	230	174	95	13	96	95	25	16.4 (13)
Terbuthylazine-2-hydroxy	6.92	3.28	212	156	95	13	86	95	25	28 (13)
Terbuthylazine-desethyl	6.98	2.81	202	146	95	13	79	95	25	13.2 (14)
Terbutryn	10.63	1.2	242	186	120	20	71	120	15	4.6 (14)
Thiabendazole	5.06	3.5	202	175	95	25	131	95	25	29.1 (18)
Thiamethoxam	2	2.58	292	211	78	10	132	78	10	21.3 (11)
Tolclofos-methyl	12.13	1.71	301	125	115	12	269	120	15	73.8 (19)

a *t_R_* = retention time.

b Δ *t_R_* = delta retention time, that is the centered retention time window.

c SRM_1_ = selected product ion for quantification.

d Frag = Fragmentor.

e CE = Collision energy.

f SRM_2_ = selected product ion for qualification.

g (%RSD) = relative standard deviation of the ratio SRM_2_/SRM_1_, calculated from mean values obtained from the matrix-matched calibration curves.
