# Supplementary material for: Efficiency of QuEChERS approach for determining 52 pesticide residues in honey and honey bees
Source: MethodsX. 2016 May 18;3:452–8. doi: 10.1016/j.mex.2016.05.005 (PMC4909158; doi:10.1016/j.mex.2016.05.005)
Supplement: Supplementary file 1 [file mmc1.docx]

SUPPLEMENTARY MATERIAL

**Effyciency of QuEChERS approach for determining 52 pesticide residues in honey and honey bees**

Pau Calatayud-Vernich^1^, Fernando Calatayud^2^, Enrique Simó ^2^, Yolanda Picó1

1*Environmental and Food Safety Research Group (SAMA-UV), Research Center on Desertification (CIDE, UV-CSIC-GV), Faculty of Pharmacy, University of Valencia, Av. Vicent Andrés Estellés s/n, 46100 Burjassot, Valencia, Spain.*

^2^*Agrupación de Defensa Sanitaria Apícola apiADS, Ctra. Montroi-Turís, 46193 Montroi, Valencia, Spain.*

**Figure S1.** Chromatograms extracted from 500 µg·L^-1^ standard of all pesticides analyzed.

| HONEY BEE | | | | | | |
| --- | --- | --- | --- | --- | --- | --- |
|  | QuEChERS | | | Solvent | | |
| Pesticide  **Table S1.** Validation data of QuEChERS and solvent approaches for honey bee matrix. LOQ, recovery (R), precision (RSD) and matrix effects of the analyzed pesticides. | LOQ  ng·g^-1^ | R ± RSD  (%) | Matrix  effects  (%) | LOQ  ng·g^-1^ | R ± RSD  (%) | Matrix  effects  (%) |
| Acetamiprid | 3.9 | 92 ± 12 | -25 | 0.8 | 56 ± 16 | -30 |
| Acetochlor | 3.9 | 95 ± 15 | -30 | 0.8 | 34 ± 18 | -45 |
| Alachlor | 3.9 | 89 ± 13 | -35 | 0.8 | 34 ± 18 | -25 |
| Atrazine | 3.9 | 91 ± 16 | -20 | 1.5 | 59 ± 17 | -30 |
| Atrazine-desethyl | 7.5 | 85 ± 17 | -28 | 1.5 | 57 ± 14 | -55 |
| Atrazine-desisopropyl | 7.5 | 89 ± 15 | -32 | 1.5 | 52 ± 14 | -52 |
| Azinphos-ethyl | 3.9 | 93 ± 12 | -15 | 0.8 | 96 ± 14 | -15 |
| Azinphos-methyl | 3.9 | 78 ± 14 | -15 | 0.8 | 91 ± 13 | 2 |
| Buprofezin | 1 | 94 ± 10 | -18 | 0.03 | 69 ± 12 | -13 |
| Carbendazim | 10 | 92 ± 10 | -35 | 2 | 58 ± 14 | -15 |
| Carbofuran | 1 | 73 ± 18 | 35 | 0.5 | 72 ± 12 | -25 |
| Carbofuran-3-hydroxy | 10 | 90 ± 15 | 10 | 2 | 64 ± 12 | -45 |
| Chlorfenvinphos | 10 | 94 ± 10 | -40 | 2 | 74 ± 14 | -30 |
| Chlorpyrifos | 1 | 95 ± 11 | -15 | 0.04 | 81 ± 18 | 1 |
| Coumaphos | 3.9 | 87 ± 12 | -10 | 0.9 | 88 ± 14 | -15 |
| Diazinon | 1 | 83 ± 15 | -30 | 0.06 | 91 ± 11 | -12 |
| Dichlofenthion | 3.9 | 87 ± 12 | -22 | 1.0 | 87 ± 11 | -35 |
| Dimethoate | 3.9 | 88 ± 12 | -27 | 0.8 | 92 ± 12 | -16 |
| Diuron | 10 | 85 ± 11 | -38 | 2 | 60 ± 16 | -23 |
| DMF | 1 | 84 ± 6 | -28 | 0.2 | 85 ± 12 | -32 |
| Ethion | 1 | 88 ± 10 | -42 | 0.2 | 91 ± 11 | -4 |
| Fenitrothion | 3.9 | 83 ± 18 | -30 | 0.8 | 83 ± 12 | -15 |
| Fenthion | 10 | 90 ± 15 | -5 | 2 | 82 ± 7 | -20 |
| Fipronil | 1 | 82 ± 8 | -19 | 0.2 | 70 ± 16 | -15 |
| Flumethrin | 3.9 | 86 ± 8 | -25 | 0.8 | 83 ± 14 | -23 |
| Fluvalinate | 1 | 93 ± 10 | -28 | 0.2 | 86 ± 15 | -15 |
| Hexythiazox | 1 | 85 ± 12 | -15 | 0.2 | 93 ± 13 | -8 |
| Imazalil | 3.9 | 81 ± 10 | -30 | 1 | 77 ± 12 | -24 |
| Imidacloprid | 1 | 91 ± 15 | -28 | 0.5 | 76 ± 11 | -33 |
| Isoproturon | 3.9 | 86 ± 10 | -35 | 0.8 | 70 ± 11 | -23 |
| Malathion | 3.9 | 88 ± 9 | -15 | 0.2 | 83 ± 12 | -5 |
| Methiocarb | 10 | 95 ± 7 | -33 | 1 | 68 ± 10 | -35 |
| Methoalachlor | 1 | 80 ± 15 | -22 | 0.12 | 76 ± 11 | -34 |
| Molinate | 10 | 86 ± 15 | -21 | 2 | 61 ± 12 | -15 |
| Omethoate | 1 | 82 ± 19 | -12 | 0.2 | 87 ± 13 | 23 |
| Parathion-ethyl | 10 | 81 ± 18 | -16 | 2 | 94 ± 12 | -7 |
| Parathion-methyl | 10 | 77 ± 15 | -18 | 2 | 91 ± 13 | -10 |
| Prochloraz | 3.9 | 96 ± 8 | -24 | 0.8 | 92 ± 12 | -14 |
| Propanil | 1 | 82 ± 8 | -38 | 0.05 | 79 ± 15 | -18 |
| Propazine | 1 | 78 ± 19 | -22 | 0.1 | 60 ± 15 | -23 |
| Pyriproxifen | 10 | 89 ± 16 | -50 | 2 | 92 ± 11 | -11 |
| Simazine | 10 | 83 ± 10 | -60 | 2 | 42 ± 18 | -56 |
| Tebuconazole | 3.9 | 91 ± 8 | -24 | 0.8 | 79 ± 14 | -25 |
| Terbumeton | 3.9 | 82 ± 10 | -33 | 0.8 | 62 ± 13 | -23 |
| Terbumeton-desethyl | 1 | 85 ± 10 | -28 | 0.1 | 51 ± 14 | -32 |
| Terbuthylazine | 3.9 | 89 ± 15 | -38 | 0.8 | 60 ± 13 | -27 |
| Terbuthylazine-2-hydroxy | 3.9 | 97 ± 10 | -40 | 1 | 92 ± 13 | -31 |
| Terbuthylazine-desethyl | 3.9 | 82 ± 10 | -38 | 1 | 90 ± 10 | -34 |
| Terbutryn | 3.9 | 87 ± 10 | -22 | 0.8 | 59 ± 14 | -43 |
| Thiabendazole | 10 | 82 ± 11 | -25 | 2 | 80 ± 10 | -22 |
| Thiamethoxam | 3.9 | 84 ± 9 | -30 | 0.8 | 81 ± 15 | -31 |
| Tolclofos-methyl | 3.9 | 90 ± 10 | -20 | 0.8 | 85 ± 17 | -15 |

| HONEY | | | | | | |
| --- | --- | --- | --- | --- | --- | --- |
| **Table S2.** Validation data of QuEChERS and SPE approaches for honey matrix. LOQ, recovery (R), precision (RSD) and matrix effects of the analyzed pesticides. | QuEChERS | | | SPE | | |
| Pesticide | LOQ  ng·g^-1^ | R ± RSD  (%) | Matrix  effects  (%) | LOQ  ng·g^-1^ | R ± RSD  (%) | Matrix  effects  (%) |
| Acetamiprid | 3.5 | 90 ± 11 | -18 | 1 | 94 ± 11 | -5 |
| Acetochlor | 2.5 | 90 ± 18 | 23 | 1 | 92 ± 9 | -8 |
| Alachlor | 2.5 | 78 ± 33 | 20 | 1 | 94 ± 11 | -15 |
| Atrazine | 3 | 80 ± 18 | -12 | 1 | 94 ± 10 | -10 |
| Atrazine-desethyl | 5 | 76 ± 19 | -15 | 2 | 78 ± 13 | -30 |
| Atrazine-desisopropyl | 6 | 89 ± 41 | -25 | 2 | 63 ± 13 | -25 |
| Azinphos-ethyl | 3 | 94 ± 11 | -8 | 1 | 90 ± 10 | 1 |
| Azinphos-methyl | 3 | 70 ± 13 | -4 | 1 | 74 ± 13 | 3 |
| Buprofezin | 1 | 94 ± 11 | -14 | 1 | 93 ± 10 | -2 |
| Carbendazim | 5 | 79 ± 18 | -35 | 5 | 82 ± 7 | -8 |
| Carbofuran | 1 | 56 ± 15 | 26 | 0.5 | 90 ± 11 | -3 |
| Carbofuran-3-hydroxy | 10 | 96 ± 28 | 15 | 2 | 80 ± 15 | 17 |
| Chlorfenvinphos | 5 | 91 ± 16 | -8 | 2 | 81 ± 12 | -15 |
| Chlorpyrifos | 0.5 | 87 ± 15 | 3 | 0.5 | 87 ± 12 | -1 |
| Coumaphos | 2 | 88 ± 13 | -12 | 1 | 91 ± 16 | -4 |
| Diazinon | 0.5 | 73 ± 22 | -10 | 0.5 | 89 ± 11 | -17 |
| Dichlofenthion | 2 | 80 ± 14 | -8 | 1 | 87 ± 13 | -21 |
| Dimethoate | 1.5 | 82 ± 21 | 15 | 1 | 42 ± 12 | -8 |
| Diuron | 5 | 80 ± 18 | -15 | 2 | 85 ± 12 | -10 |
| DMF | 1 | 85 ± 10 | -22 | 0.5 | 85 ± 21 | -24 |
| Ethion | 0.5 | 77 ± 23 | -14 | 0.5 | 86 ± 13 | -34 |
| Fenitrothion | 2 | 30 ± 16 | 4 | 2 | 88 ± 11 | -15 |
| Fenthion | 5 | 79 ± 22 | 12 | 3 | 97 ± 16 | -8 |
| Fipronil | 1 | 81 ± 8 | -16 | 0.5 | 80 ± 10 | -14 |
| Flumethrin | 3 | 88 ± 12 | -21 | 0.5 | 78 ± 13 | -15 |
| Fluvalinate | 1 | 95 ± 10 | -25 | 0.1 | 78 ± 12 | -10 |
| Hexythiazox | 0.5 | 78 ± 23 | -2 | 0.5 | 89 ± 11 | -15 |
| Imazalil | 1 | 82 ± 14 | 25 | 2 | 92 ± 12 | -5 |
| Imidacloprid | 0.5 | 84 ± 25 | 24 | 0.5 | 82 ± 9 | -7 |
| Isoproturon | 2 | 82 ± 14 | -25 | 1 | 97 ± 10 | -15 |
| Malathion | 3 | 70 ± 29 | -4 | 1 | 92 ± 14 | -1 |
| Methiocarb | 5 | 91 ± 7 | -12 | 2 | 80 ± 11 | -4 |
| Methoalachlor | 0.5 | 69 ± 23 | 15 | 0.5 | 75 ± 11 | -15 |
| Molinate | 5 | 68 ± 17 | -12 | 1 | 91 ± 10 | -8 |
| Omethoate | 0.5 | 63 ± 24 | 33 | 0.2 | 83 ± 12 | -12 |
| Parathion-ethyl | 5 | 84 ± 18 | -14 | 2 | 89 ± 8 | -8 |
| Parathion-methyl | 5 | 82 ± 14 | -17 | 2 | 93 ± 8 | -5 |
| Prochloraz | 2 | 81 ± 15 | -12 | 1 | 91 ± 10 | -6 |
| Propanil | 1 | 82 ± 26 | -22 | 0.2 | 77 ± 13 | -15 |
| Propazine | 1 | 78 ± 13 | -15 | 0.3 | 90 ± 9 | -5 |
| Pyriproxifen | 5 | 89 ± 12 | -12 | 1 | 77 ± 11 | -14 |
| Simazine | 10 | 79 ± 14 | -60 | 2 | 75 ± 12 | -17 |
| Tebuconazole | 2 | 85 ± 18 | 50 | 1 | 92 ± 8 | -5 |
| Terbumeton | 2 | 83 ± 9 | -21 | 1 | 95 ± 9 | -8 |
| Terbumeton-desethyl | 0.5 | 82 ± 11 | -25 | 0.4 | 90 ± 10 | -26 |
| Terbuthylazine | 2 | 74 ± 25 | -27 | 1 | 89 ± 12 | -12 |
| Terbuthylazine-2-hydroxy | 3 | 99 ± 30 | -24 | 1 | 61 ± 15 | -8 |
| Terbuthylazine-desethyl | 2 | 82 ± 13 | -15 | 1 | 80 ± 13 | -32 |
| Terbutryn | 3 | 74 ± 26 | -15 | 0.4 | 83 ± 11 | -15 |
| Thiabendazole | 5 | 74 ± 15 | 22 | 2 | 86 ± 11 | -12 |
| Thiamethoxam | 3 | 84 ± 11 | -25 | 1 | 86 ± 12 | -24 |
| Tolclofos-methyl | 3.9 | 89 ± 13 | -15 | 1 | 86 ± 11 | -15 |
